# Assessing Motor Cortical Activity: How Repetitions Impact Motor Execution and Imagery Analysis

**DOI:** 10.1111/psyp.70090

**Published:** 2025-06-10

**Authors:** Marta Borràs, Sergio Romero, Leidy Y. Serna, Joan F. Alonso, Alejandro Bachiller, Miguel A. Mañanas, Mónica Rojas

**Affiliations:** ^1^ Biomedical Engineering Research Centre (CREB), Department of Automatic Control (ESAII) Universitat Politècnica de Catalunya (UPC) Barcelona Spain; ^2^ CIBER de Bioingeniería, Biomateriales y Nanomedicina (CIBER‐BBN) Madrid Spain; ^3^ Institut de Recerca Sant Joan de Déu Barcelona Spain

**Keywords:** electroencephalography (EEG), event related desynchronization (ERD), LORETA, motor execution (ME), motor imagery (MI), motor‐related cortical activity, motor‐related cortical potential (MRCP)

## Abstract

The study of motor‐related cortical activity is crucial for analyzing brain behavior during motor execution (ME) and imagery (MI). Improving motor learning and recovery in patients with motor disorders involves both ME and MI. Although ME and MI share the same motor brain network, multiple studies show differences in motor‐related cortical activity regarding amplitude, timing, and fatigue. Movement‐related cortical potentials (MRCPs) and event‐related desynchronization (ERD) are key motor‐related cortical activities in time and frequency domains. These are used to characterize and monitor neuromotor pathologies through averaging techniques. However, a sufficient number of trials is needed for cortical activity averaging, which may prolong tasks and induce patient fatigue, potentially affecting the results. The aim of this work was to analyze the effect of the number of trials on MRCPs and ERD mu and beta bands during upper‐limb movements: elbow flexion/extension, forearm pronation/supination, and hand open/close. Differences between ME and MI were assessed using Monte Carlo analysis of motor‐related cortical features, scalp topography activity, and low‐resolution electromagnetic tomography (LORETA). The impact of reduced trials varied by movement and feature. Certain differences between ME and MI became statistically nonsignificant with fewer trials. Hand opening/closing and ERD in the mu band were most sensitive to reduced trials. Results were supported by topographic maps and LORETA images, linking reduced trials to increased intersubject variability. These findings highlight the need for an optimal number of trials to ensure reliable outcomes.

## Abstract 2

Event‐related potentials are essential for analyzing brain activity during motor execution (ME) and imagery (MI), both vital for motor learning and rehabilitation. Recording protocols can be lengthy and tedious. This study analyzed the impact of limited repetitions on cortical activity during ME and MI of upper‐limb movements, emphasizing the importance of optimizing trial numbers for reliable results and reduced variability. Generally, fewer than 50 trials made it harder to differentiate certain motor‐evoked features between groups.

## 1 Introduction

Motor‐related cortical activity is related not only to motor execution (ME) but also to imagined movement, known as motor imagery (MI). MI is the cognitive process of internally simulating a movement without actual execution, involving the activation of motor‐related brain regions without producing any overt physical action (Jeannerod 2001). Although the role of MI in brain‐computer interface (BCI) technologies is well established, particularly in the rehabilitation of motor impairments following neurological damage (Mane et al. 2020; Wang et al. 2024; Zhang et al. 2024), MI has also demonstrated its ability as a therapy to facilitate brain plasticity by reconfiguring cortical organization and enhancing functional restoration (Mulder 2007; Zich et al. 2015). In recent decades, both visual and kinesthetic MI have been recognized as valuable for enhancing motor learning and recovery in patients with motor impairments (Adams et al. 2018; Charbonnier et al. 2024; Guillot and Debarnot 2019). Specifically, kinesthetic MI engages motor planning and sensorimotor processes, generating brain activity patterns similar to actual movement execution (Debarnot et al. 2021; Lomelin‐Ibarra et al. 2022).

Despite several works demonstrating the activation of overlapping brain regions in both ME and MI (Jeong et al. 2020; Ofner et al. 2017; Van der Lubbe et al. 2021; Wairagkar et al. 2021), the similarities and differences between these two processes remain unclear in current research. The overlapping areas vary across studies, but they commonly comprise the premotor cortex, supplementary motor area (SMA), superior parietal, and inferior parietal cortex (Claflin et al. 2015). However, recent works agreed that despite ME and MI activating similar brain areas during the preparation stage, they differed during the execution stage: MI involves inhibiting the motor response, whereas ME relies on visual sensory feedback (Angelini et al. 2015; Gerardin et al. 2000; Lotze and Halsband 2006; Meng et al. 2018; Van der Lubbe et al. 2021). Supporting this distinction, Syrov et al. (2024) observed differences in lateralized brain activity peaks between ME and MI using a single‐trial analysis. Their findings indicate that activity accumulation begins prior to both ME and MI responses; however, lateralization only occurs when an actual movement is required. Moreover, when MI tasks were not learned, the magnitude of imagery‐induced activity in these areas has been shown to be much lower than the one during actual movement (Lotze and Halsband 2006; Miller et al. 2010; Nascimento et al. 2006). Similarly, Guillot et al. (2008) reported individual differences in functional neuroanatomical networks associated with motor imagery abilities. Beyond these distinctions, reduced brain activity has been noted not only during MI but also in paradigms involving movement inhibition, such as action cancelation and motor attempts, in contrast to actual movement execution (Galdo‐Alvarez et al. 2016). Another notable difference between ME and MI relates to the nature of fatigue they induce. While ME can lead to physical fatigue, MI predominantly results in mental fatigue (Guillot et al. 2009; Jeannerod 2001). Overall, although both processes engage the same motor brain network, they differ in activation levels, sensory feedback mechanisms, timing of neural engagement, and the type of fatigue they predominantly elicit.

Motor‐related cortical activity is the brain activation in response to specific events and is usually recorded using an electroencephalography (EEG) technique (Kropotov 2016; Sur and Sinha 2009). Event‐related potentials (ERPs) that are linked to either the execution or imagery of movement are referred to as movement‐related cortical potentials (MRCPs). They can be distinguished into self‐initiated potentials, such as the readiness potential or Bereitschafts potential, and stimulus‐related potentials (Nann et al. 2019; Sur and Sinha 2009; Teodoro et al. 2020). MRCPs are slow potentials related to motor readiness and appear before the movement onset irrespective of whether a stimulus is present before the action (Colebatch 2007; Schurger et al. 2021). The primary motor area, the SMA, and the premotor cortex are the primary regions where significant MRCP activity is observed (Colebatch 2007; Rektor et al. 2003). In contrast, movement‐related changes in brain activity can also be observed in the frequency domain. One example is event‐related desynchronization (ERD), which refers to alterations in the synchronization of cortical rhythms associated with the planning and execution/imagery of a movement, as well as their subsequent recovery (Jeon et al. 2011; Neuper and Pfurtscheller 2001; Pfurtscheller and Lopes da Silva 1999). In particular, the mu (8–13 Hz) and beta (14–30 Hz) frequency bands, in which ERD reactions are most commonly studied, have been widely investigated (Aoh et al. 2019; Jeon et al. 2011; Li et al. 2018; McFarland et al. 2000).

Both MRCP and ERD features offer valuable insights into the functional organization of the motor cortex and provide a means of evaluating the responsiveness of the motor system to external stimuli. Motor‐related cortical activity features have been shown to be relevant when characterizing pathologies or following a rehabilitation process of motor recovery. In particular, MRCP has been used to characterize neuromotor disabilities such as stroke, amyotrophic lateral sclerosis (ALS), motor stereotypies, among others (Bizovičar et al. 2013; Chen et al. 2022; Gong et al. 2013; Houdayer et al. 2014; Platz et al. 2000; Westphal et al. 1998). On the other hand, ERD has also been conducted on patients with motor impairments such as Parkinson's disease, stroke, ALS, dystonia, and other conditions (Heida et al. 2014; Hess et al. 2020; Hosni et al. 2019; Platz et al. 2000; Stępień et al. 2011). Additionally, the MRCP and ERD characteristics have also been examined to monitor the motor rehabilitation progress of patients with certain motor disabilities, as well as to evaluate the efficacy of motor rehabilitation programs, helping clinicians tailor interventions to meet the individual needs of patients and optimize their recovery (El Nahas et al. 2022; Fong et al. 2021; Honda et al. 1997).

Moreover, MRCP features are often represented spatially either as topographic maps, depicting the distribution of potentials across the scalp (Chen et al. 2018; Nann et al. 2019), or as tomographic maps, which provide a more accurate approximation of MRCP localization by estimating the brain's electrical sources (Lamm et al. 2005; Michel and He 2019; Pascual‐Marqui et al. 2011, p. 20). EEG‐based source localization has been widely applied to improve the characterization of motor impairments, to provide insights for BCI applications, and to enhance EEG classification (Courellis et al. 2017; Muhamed et al. 2016; Painold et al. 2011; Santos et al. 2021; Xu and Li 2023).

Motor disabilities caused by brain injury and/or diseases can affect different parts of the body depending on the brain region affected. Consequently, patients may experience difficulties when performing different types of movements. Although motor‐related brain activity exhibits common features across movement types within the same limb, previous studies have reported variations in MRCPs and/or ERD/ERS patterns (Ofner et al. 2017, 2019; Vuckovic and Sepulveda 2008; Yong and Menon 2015). Therefore, investigating the consistency of these neural patterns across movement types is relevant for understanding their robustness and generalizability.

MRCPs as well as ERD can be better obtained by averaging a given number of trials or repetitions (Chen et al. 2018; Pfurtscheller and Lopes da Silva 1999; Schurger et al. 2021; Visani et al. 2019). The task of recording a large number of trials can be tedious for patients and can lead to physical and/or mental fatigue, which can produce changes in motor‐related cortical activity (Ali A. Abdul‐latif et al. 2004; Boksem et al. 2005; Jacquet, Lepers, et al. 2021; Jacquet, Poulin‐Charronnat, et al. 2021). This is especially crucial in experiments with children or when several tasks and experiments are performed by patients in the same session, which can be extended excessively. Several studies have analyzed the impact of the number of trials on the motor‐related cortical activity waveforms (Borràs et al. 2022; Boudewyn et al. 2018; Thigpen et al. 2017). These works globally estimate the appropriate number of repetitions as a function of the techniques used in each study. For instance, 50 trials were suggested to obtain reliable results during upper movements in terms of MRCP and ERD (Borràs et al. 2022). In the case of MI, this study has not been carried out yet. It is known that in spite of MI involves similar involve the same motor brain plan and mechanisms as ME, the magnitude and intensity of the former are lower than the latter. Therefore, it is important to examine the effect of trial number on both ME and imagery to fully understand the similarities and differences between these two processes on the dynamics of brain activity during movement planning and readiness.

This study aimed to investigate exhaustively the effect of trial number on motor‐related brain activity during ME and MI tasks. Specifically, the study focused on analyzing MRCPs and ERD mu and beta features during upper‐limb movements, including elbow flexion and extension, forearm pronation and supination, and hand open and close. Neural processes underlying motor planning and execution, and how these processes differ between ME and MI, were assessed by means of time‐ and frequency‐domain averaged features, scalp topography activity, and low‐resolution electromagnetic tomography (LORETA). We hypothesize that the differences between ME and MI may diminish as the number of trials decreases. Specifically, we expected this effect to manifest in MRCP and ERD amplitudes, as well as in their spatial distribution on the scalp (topography) and in the brain (tomography). Prior studies have demonstrated that reducing the number of trials compromises the accuracy of brain activity analyses, leading to amplitude variations and a more diffuse localization of neural signals (Borràs et al. 2022; Sieluzycki et al. 2021; Thigpen et al. 2017).

## 2 Methods

### 2.1 Subjects

The EEG data analyzed in this study were collected from an open dataset (Ofner et al. 2017). Fifteen healthy subjects (9 women) comprised the dataset, fourteen of whom were right‐handed. The average age was 27 years, ranging from 22 to 40 years (standard deviation: 5 years). The protocol was approved by the ethics committee of the Medical University of Graz, and all volunteers signed an informed consent.

### 2.2 Experimental Design

Two recording sessions were performed in two different days (within 1 week). On the first day, participants performed a set of predefined movements (ME). In the second session, they were instructed to imagine the same movements without executing them (MI). To enhance kinesthetic MI, participants completed one ME run immediately before the MI session.

**FIGURE 1:**
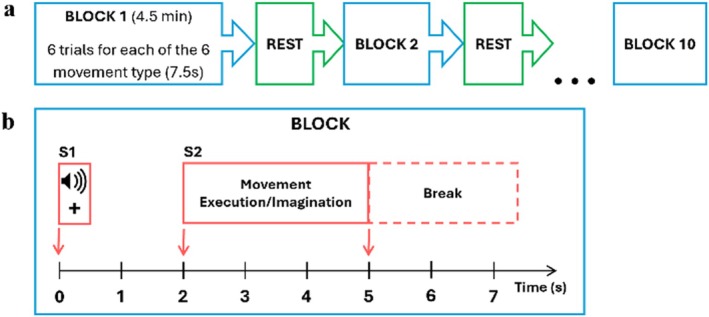
(a) Block diagram. (b) Paradigm diagram: S1 denotes the warning stimulus (auditory beep and visual cross), and S2 represents the movement stimulus (image of the required movement). The same timeline was used for both ME and MI sessions on separate days within the same week.

Participants sat on a chair, in front of a computer screen, with their arms fully supported by an exoskeleton to reduce muscle fatigue. During data recording, all participants had to perform or imagine six different movements with the right upper limb: elbow flexion and extension, forearm supination and pronation, and hand open and close. Subjects had to follow an S1‐S2 contingent negative variation (CNV) paradigm: at second 0, a primary attention stimulus (S1) appeared on the computer screen (as a cross) together with a beep sounded; afterwards, at second 2, a cue with movement type instructions (S2) was presented on the computer screen, and subjects had to perform/imagine the movement (Figure 1b). After each movement, participants had to return to the initial position: hand half open and lower arm extended at 120° in a neutral position. The choice of movement that subjects were required to perform was randomized for each trial, with an equal number of repetitions for each type of movement.

In both ME and MI sessions, 60 trials per movement were recorded, distributed in ten blocks (of 6 trials per movement) in order to reduce mental and physical fatigue. Each recording session lasted approximately 1 h, consisting of 10 blocks of 4.5 min each (Figure 1a). In this study, movement classes were pooled together into three groups as performed in (Borràs et al. 2022): elbow flexion/extension, forearm supination/pronation, and hand open/close. Thus, a total of 120 trials per movement class were obtained.

### 2.3 Data Recording

The dataset was acquired using 61 active electrodes of EEG and 3 electrooculography (EOG) channels. EEG electrodes were placed on the scalp according to the international 5/10 system and referenced to the right mastoid (ground on AFz). Four 16‐channel amplifiers (g.tec medical engineering GmbH, Austria) were used to record data at a sample frequency of 512 Hz. Afterwards, EEG and EOG signals were analogically filtered using an 8th order Chebyshev band‐pass filter from 0.01 Hz to 200 Hz. In addition, accelerometers attached to the exoskeleton and grip pressure sensors from the Data Glove (5DT, USA) recorded the position of the arm and hand of the volunteer in order to monitor physical movements and thus determine movement onsets.

### 2.4 Data Processing

EEG data from both ME and MI recordings was preprocessed following the procedure used in (Borràs et al. 2022). Firstly, a band‐pass filter was applied to EEG and EOG raw channels from 0.3 to 100 Hz. Secondly, EEG and EOG data was decomposed into source components using a second order blind identification (SOBI) algorithm (Belouchrani et al. 1997) in order to reduce ocular contamination from EEG signals. These ocular‐related sources were automatically identified by means of frequency and scalp topography features of the obtained components (Romero et al. 2008). Ocular‐artifact free EEG signals were reconstructed from the remaining source components.

For identifying artefactual trials, they were considered between −0.5 s and 7 s, with the S1 stimulus occurring at 0 s. Subsequently, EEG channels that were identified as outliers in terms of trial amplitude and kurtosis were automatically labeled as artifactual channels and rejected. We marked as outliers those trials exhibiting amplitude and kurtosis values above a threshold, which was defined as five times the standard deviation of each one. For each subject and movement class, trials containing more than 25% of EEG channels labeled as artifacts were also excluded from the analysis. Finally, EEG data was rereferenced to a common average reference, excluding the rejected channels, in order to emphasize the local activity associated with the movement (Platz et al. 2000).

### 2.5 Movement Onset Identification

The movement onset of each ME trial was detected by using the data from the accelerometers and sensors of the glove and the exoskeleton (Borràs et al. 2022). Data showed noisy upward or downward steps depending on the exercise. As a result, once the derivative was thresholded and a preliminary estimation of the desired landmarks was obtained, the values were further refined by identifying the first local minimum or maximum that appeared just before the step. These refined values were considered as the onset times. Movement onsets of MI trials were estimated by averaging movement onsets obtained from ME trials separately for each subject and movement class (Ofner et al. 2017).

### 2.6 Motor‐Related Cortical Potential (MRCP) Analysis

In this study, MRCP is a stimulus potential induced by the S1‐S2 paradigm. This potential is also referred to as the CNV, and it consists of both early and late components. The late component involves the motor preparation and planning associated with the motor response required by the imperative stimulus S2 (Schurger et al. 2021). MRCPs are commonly characterized by the peak amplitude of the wave, which typically occurs shortly before the movement onset (Chen et al. 2018; Colebatch 2007; Schurger et al. 2021). To determine the MRCPs, EEG potentials were averaged using five‐second windows (from −2.5 to 2.5 s relative to the movement onset) after applying a 4th‐order Butterworth band‐pass filter between 0.3 to 3 Hz to the preprocessed EEG signals. The feature MRCP peak was calculated at the Cz channel by averaging the amplitude values within a window of 200 ms, centered at the maximum peak identified within the 500 ms window before the onset of the movement (Borràs et al. 2022).

### 2.7 Event‐Related Desynchronization (ERD) Assessment

ERD refers to a decrease in the power of EEG activity, in relation to a reference period, that occurs when a subject performs or imagines a movement (Jeon et al. 2011; Pfurtscheller and Lopes da Silva 1999). ERD is thought to reflect the suppression of cortical activity in the motor cortex associated with movement planning (Pfurtscheller and Lopes da Silva 1999). In this study, ERD was computed in the most commonly analyzed frequency bands: mu (8–13 Hz) and beta (14–30 Hz) (Aoh et al. 2019; Jeon et al. 2011; Li et al. 2018). The EEG signal was first bandpass‐filtered within these frequency ranges, and the power was obtained by squaring the filtered signal. The power time series was then segmented into individual trials, covering a time window from −2.5 to 5.5 s relative to movement onset. Then, the average power across all trials was computed. ERD was calculated as the percentage change in power relative to a fixed reference window (from −1.5 to −1 s before movement onset, denoted by *Rw*), following the classical approach described by Pfurtscheller and Lopes da Silva (1999) (see Equation 1).

(1) $$\mathrm{ERD}=\left(\left({\frac{1}{\text{Ntrials}}}^{*}\sum_{i=1}^{\text{Ntrials}}\text{EEGtrial}{\left(i\right)}^{2}\right)-\mathrm{Rw}\right)*\frac{100}{\mathrm{Rw}}$$

A common feature used to characterize the ERD pattern is the minimum ERD amplitude (Aoh et al. 2019; Visani et al. 2019). The calculation of the ERD min feature was performed by averaging the ERD waveform within a 200 ms window centered at the movement onset (Borràs et al. 2022).

In contrast to the MRCP peak, with a higher value predominantly located at Cz, the mu and beta ERD exhibited a widespread distribution across the scalp, involving bilateral central foci with a more prominent presence on the contralateral hemisphere of the movement. Thus, two regions of interest (ROIs) were defined, considering the channels with higher ERD consistently for mu (channels CCP3h, CP1, CP3, CPP3h, P3 and P1) and beta (channels C3, C1, CCP3h, CCP1h, CP3, CP1, CPP3h and CCP1h) bands (McFarland et al. 2000).

### 2.8 Low‐Resolution Electromagnetic Tomography (LORETA)

The standardized low‐resolution brain electromagnetic tomography (sLORETA) was used to estimate the intracranial current source densities associated with the MRCP generation (Pascual‐Marqui 2002). sLORETA uses EEG scalp voltages to compute a 3D intracerebral current density distribution. The basic principle behind the sLORETA inverse solver is the assumption that the spatial gradient of voltage changes in a consistent manner, and thus only the most evenly distributed source magnitude is chosen. In this study, the solution space was restricted for the cortical gray matter and hippocampus, resulting in a final set of 6239 voxels with a spatial resolution of 0.125 cm^3^. To identify the brain electrical sources responsible for MRCP, sLORETA images were averaged within the time interval of −100 to 100 ms around the MRCP peak.

We restricted LORETA analysis to MRCP due to their sustained voltage gradients and time‐locked cortical activation patterns, which align with LORETA's quasi‐static source modeling. In contrast, ERD reflects transient spectral network dynamics, which require time‐frequency decomposition for capturing oscillatory interactions (Bradley et al. 2016; van Vliet et al. 2018).

### 2.9 Impact of the Number of Repetitions

To derive the average motor‐related cortical activity obtained with a specific number of trials, trial subsets were selected using a Monte Carlo approach, which involved a series of random selections of repetitions for each movement class. Specifically, 1000 subsets of randomly selected trials were conducted for each subject and movement class, wherein 10 random trials were successively removed until only 20 trials remained in each subset. These subsets were then used to calculate the average motor‐related cortical activity. Thus, each Monte Carlo selection included trial subsets, with the number of trials ranging from 110 to 20 and decreasing by 10 trials for each subset, for each movement class. The purpose of this procedure was to perform a robust analysis of the effect of a reduced number of trials on obtaining the motor‐related cortical activity for each subject, movement class, and for both ME and MI.

### 2.10 Statistical Study

Statistical differences between ME and MI were assessed in a scenario where the number of trials used for the motor‐related cortical activity averaging was reduced. Repeated‐measures ANOVAs (rmANOVA) with a Greenhouse–Geisser correction were carried out for evaluating statistical motor‐related cortical activity changes for different conditions (execution or imagery) and movement classes. Paired *t*‐tests were applied for each Monte Carlo subset and movement class for each feature value (MRCP max and mu and beta ERD min). Paired *t*‐tests were also applied at each electrode to investigate statistical variations in topographic distributions of MRCP and ERD mu and beta. The significance level was set to 5%. Furthermore, to investigate statistical differences between source generators of MRCPs for ME and MI, paired *t*‐tests were conducted on log‐transformed sLORETA current density values at voxels that constitute the Brodmann Area 6 (BA6), as this region plays an essential role in the planning, preparation, and execution of voluntary movements (Hanakawa et al. 2002; Picard and Strick 1996). In order to correct for multiple comparisons in the sLORETA images, a nonparametric test using the principles of randomization and permutation was applied (Holmes et al. 1996).

Statistical analysis was conducted using IBM SPSS Statistics 30.

## 3 Results

The automatic artifact procedure based on blind source separation identified an average of 2.2 ± 1.0 (mean ± std) ocular‐related sources for the ME group, and a total of 2.1 ± 0.7 for MI. The percentage of artifactual trials excluded from the study was, on average, around 6%. Moreover, no significant differences were found in the number of rejected trials between ME and MI, as well as between movement classes. To evaluate potential intersession variability, we analyzed baseline power differences between ME (first session) and MI (second session). The analysis showed no significant differences between sessions.

**FIGURE 2:**
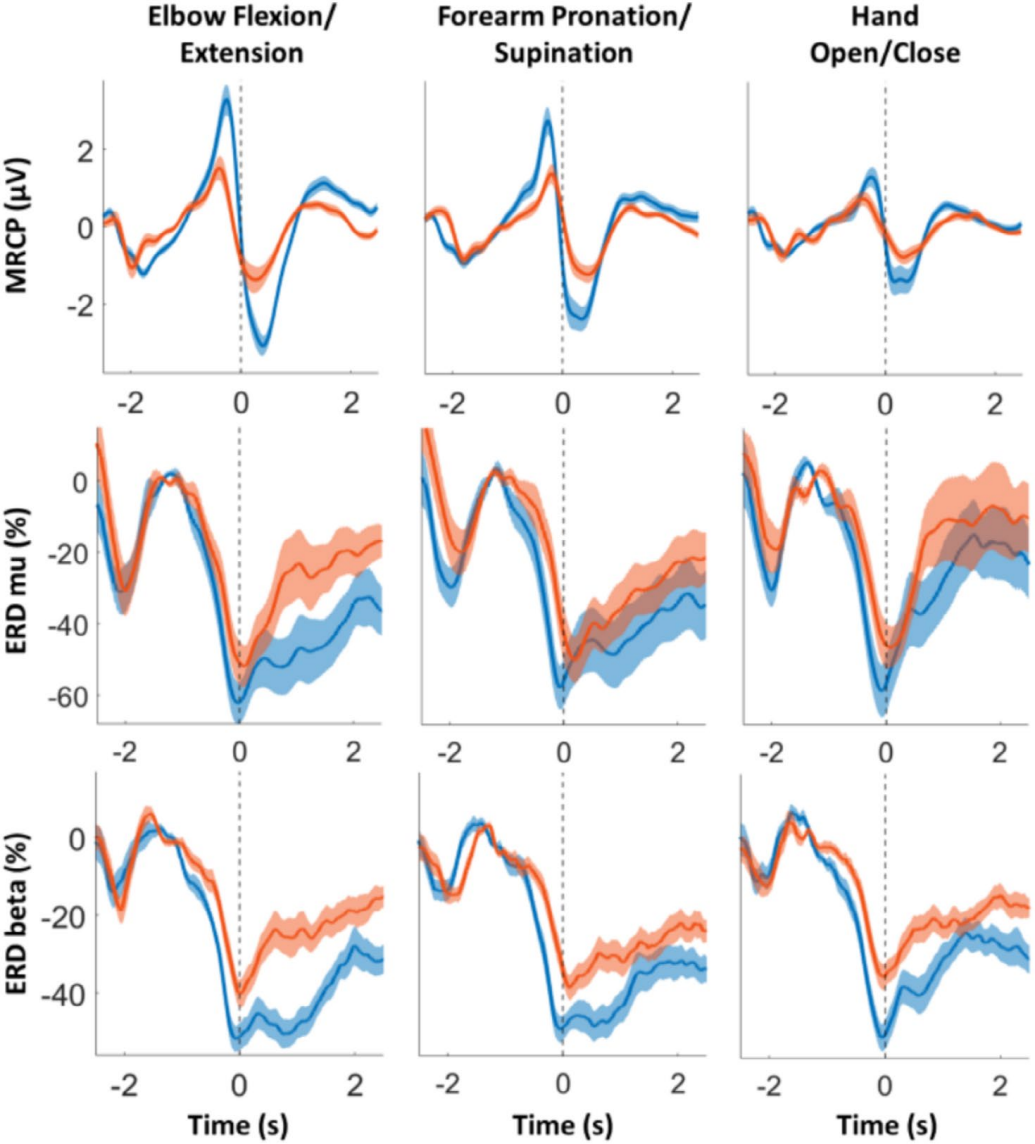
Time course of averaged motor‐related cortical activity considering all subjects and the whole 120 trials for ME (blue) and MI (red) datasets, and each feature and movement class. Standard error of the mean (SEM) within all subjects was represented as a shadow around the amplitude. MRCP was calculated at Cz and ERD/ERS in their corresponding ROIs.

Grand‐mean average motor‐related cortical activity (MRCP and ERD waveform in mu and beta bands) for both ME and MI groups is presented in Figure 2. Both MRCP and ERD were aligned to the actual and virtual movement onsets for the ME and MI groups, respectively. Differences in amplitude values between ME and MI, and between movement classes were revealed. For instance, the MRCP amplitude was greatest for elbow flexion/extension and least for hand opening/closing. MRCPs were consistently more prominent during ME than MI across all movement classes, as expected (flexion/extension and pronation/supination: *p* < 0.001; hand open/close: *p* < 0.05).

In terms of ERD waveforms, the maximum desynchronization values were obtained just at the movement onset. In the mu band, ERD min values were slightly lower for ME compared to MI across all movement classes (see Figure 2) (*p* < 0.05). On the other hand, desynchronization in the sensorimotor beta rhythm was significantly greater for ME compared to MI (*p* < 0.001). Furthermore, mu and beta ERD waveforms showed clear differences between ME and MI after the onset of the movement, likely due to visual feedback from actual movements, which is not present in MI. Additionally, a transient ERD rebound can be observed in all ERD waveforms before movement onset, which is likely a neurophysiological response to the warning stimulus.

Motor feature values were inspected by a 3‐way rmANOVA with the factors Feature (MRCP peak, and ERD min in the mu and beta bands), movement class (flexion/extension, pronation/supination, and hand opening/closing), and condition (execution and imagery) for all trials (Table 1). Significant main effects were found for Feature (*F* [2, 28] = 65.72, *p* < 0.001), movement class (*F* [2, 28] = 8.32, *p* < 0.002), and Condition (*F* [1, 14] = 33.90, *p* < 0.001). The interaction between the Feature × Condition (*F* [2, 28] = 22.27, *p* < 0.001) indicates that there are statistical differences in the features (MRCP peak, ERD in mu and beta bands) depending on the condition (ME and MI), as shown in Figure 2. Moreover, a significant Feature × Movement interaction was found (*F* [4, 56] = 5.09, *p* < 0.006), related to the different feature values for the three movement classes. No other significant interaction effects were observed.

**TABLE 1:** Tests of within subjects effects.

Factors	3‐way rmANOVA results
Feature	*F* (2, 28) = 65.72, *p* < 0.001
Movement class	*F* (2, 28) = 8.32, *p* < 0.002
Condition	*F* (1, 14) = 33.90, *p* < 0.001

Interactions	3‐way rmANOVA results
Feature × condition	*F* (2, 28) = 22.27, *p* < 0.001
Feature × movement class	*F* (4, 56) = 5.09, *p* < 0.006

**FIGURE 3:**
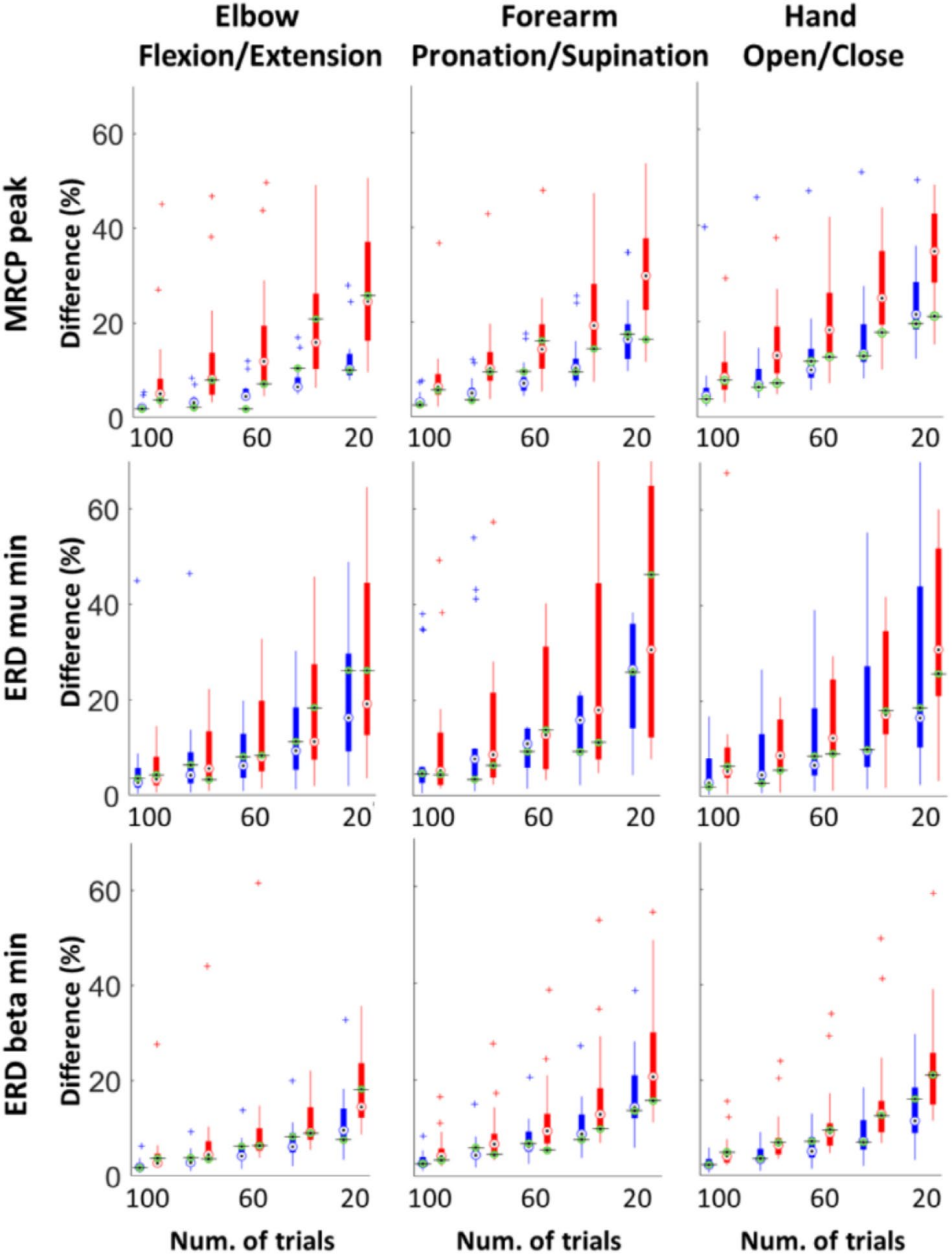
Boxplots of the differences between averaging 120 trials and a reduced number of trials for ME (blue) and MI (red) datasets, and each feature and movement category. The median difference obtained using chronological trial selection is indicated in green.

Figure 3 illustrates the impact of trial reduction on the motor features (MRCP peak and ERD min in mu and beta) for both ME and MI. Absolute percentage differences between averaging 120 trials and a reduced number of trials were calculated for each Monte Carlo subset, movement class, and subject. Average differences between all the Monte Carlo subsets for each movement class were presented as boxplots, showing the median and the 25th and 75th percentiles between subjects. Boxplots showed a clear increment of the absolute average differences as the number of trials employed for motor‐related cortical activity averaging decreased. This trend was similar for both ME and MI, although the difference values for MI were higher than those for ME, especially in MRCP max, showing that MI is more sensitive to the reduction of trials number. On the other hand, even when reduced to a set of only 20 trials, the ERD beta min displayed differences lower than 20%, being the most robust feature to trials number reduction. As the number of trials decreased, a more spread distribution was observed across subjects for all movement classes, especially in ERD mu min, and being particularly larger in the MI condition. The increase in variability for both ME and MI, as the number of trials decreases, makes it more challenging to detect statistical differences between them.

**FIGURE 4:**
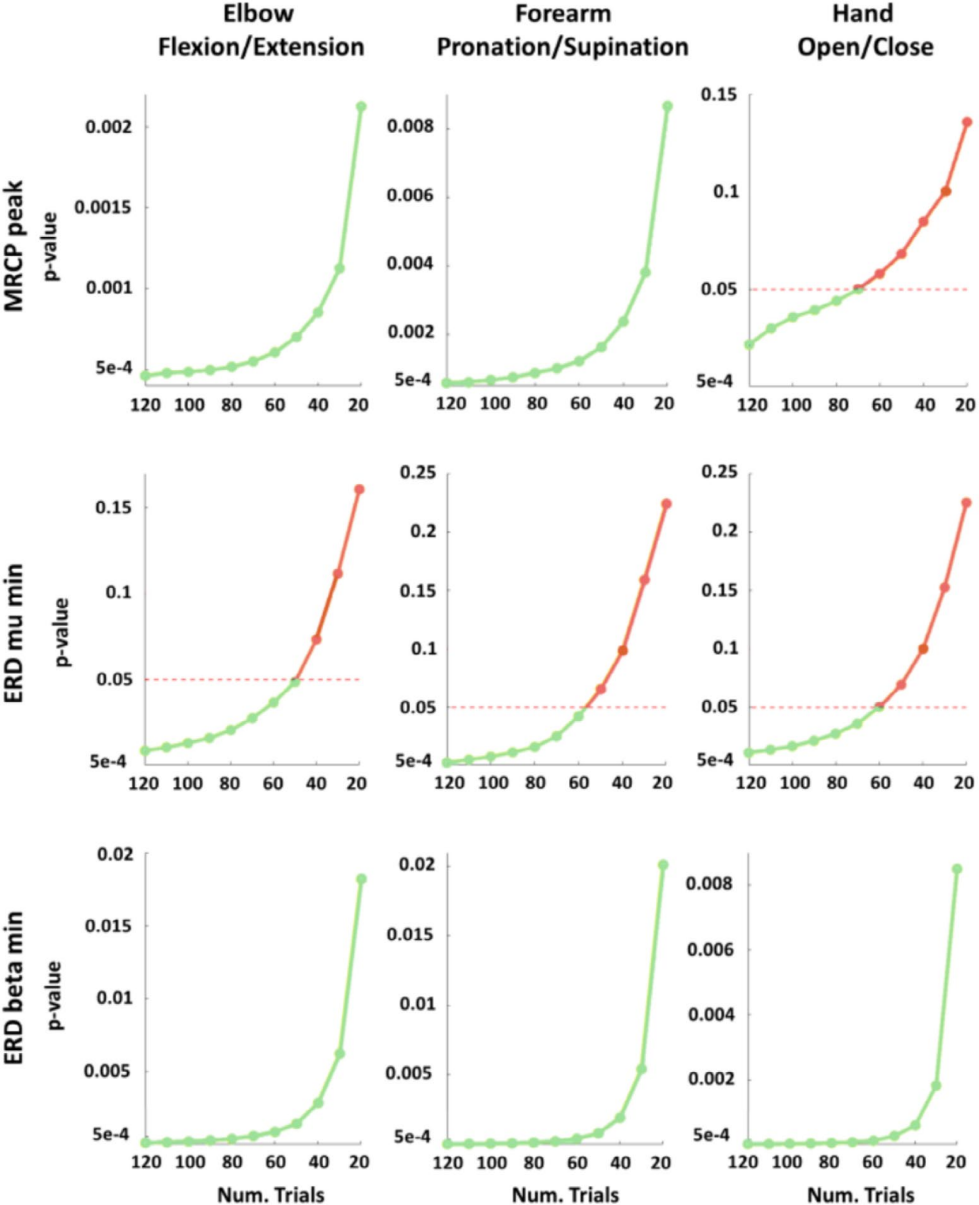
Averaged *p*‐values across all Monte Carlo subsets corresponding to the statistical paired *t*‐test between motor feature values obtained for ME and MI, for each joint movement. Green lines indicate statistically significant values (*p* ≤ 0.05), while red lines indicate non‐significant values (*p* > 0.05).

When averaging the entire 120 trials, statistically significant differences (*p* < 0.05) were found in all motor features between ME and MI for all movement classes, as expected. However, these differences were lost for certain features and movement classes when the number of trials is reduced. Figure 4 shows, for each feature and movement class, the averaged *p*‐values obtained across all Monte Carlo subsets from the statistical paired *t*‐test between motor features corresponding to ME and MI. In the case of MRCP peak, statistical differences were observed between ME and MI for elbow flexion/extension and forearm pronation/supination, even when considering only 20 trials for averaging. Nonetheless, for hand opening/closing, significance between execution and imagery was lost when considering fewer than 70 trials. The ERD beta min was the best motor feature to distinguish between all executed and imagined joint movements because it was statistically significant even for a very limited number of trials.

**FIGURE 5:**
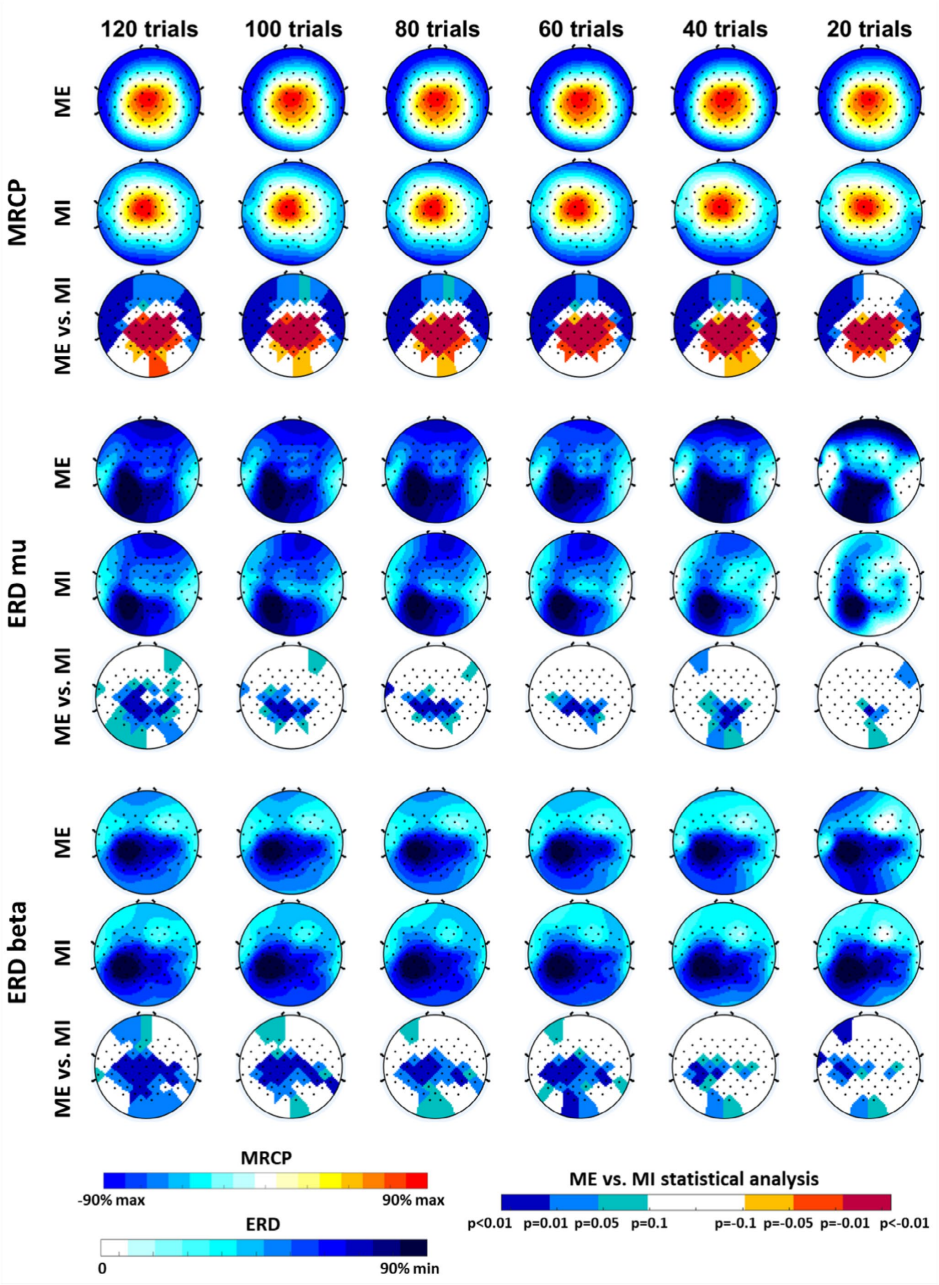
Topographic maps of ME and MI datasets and their statistical difference, averaged for all subjects and plotted for elbow flexion/extension, for each feature (MRCP, ERD mu and beta) and different number of trials. Color scales are different for MRCP and ERD features (see Figure 2 to check the minimum/maximum values), and also for ME versus MI topographic maps.

On the other hand, topographic maps were obtained to analyze the scalp distribution of the motor features (MRCP peak and ERD min in the mu and beta bands) for both ME and MI datasets. The statistical differences between them were also assessed as a function of the number of trials employed for motor‐related cortical activity averaging. Figure 5 shows an example of the topographic distributions for the flexion/extension movement, considering the first chronologically ordered trials (pronation/supination and open/close topographic maps showed similar results). Comparable results were obtained for other random trial subsets and movement classes. For both ME and MI, the average location of the motor features, especially the MRCP peak, remained consistent regardless of trial reduction. The scalp distributions for imagery movements were comparable to those of physical movements, with a slightly more anterior location for the MRCP peak and equivalent contralateral activation for ERD in both mu and beta bands. However, statistical differences were observed between ME and MI. They remained nearly regular for the MRCP peak feature across the different numbers of trials because of consistent higher values in ME than in MI, whereas statistical disparities for the ERD features gradually vanished as the number of trials reduced, possibly due to increased variability (see Figure 5).

**FIGURE 6:**
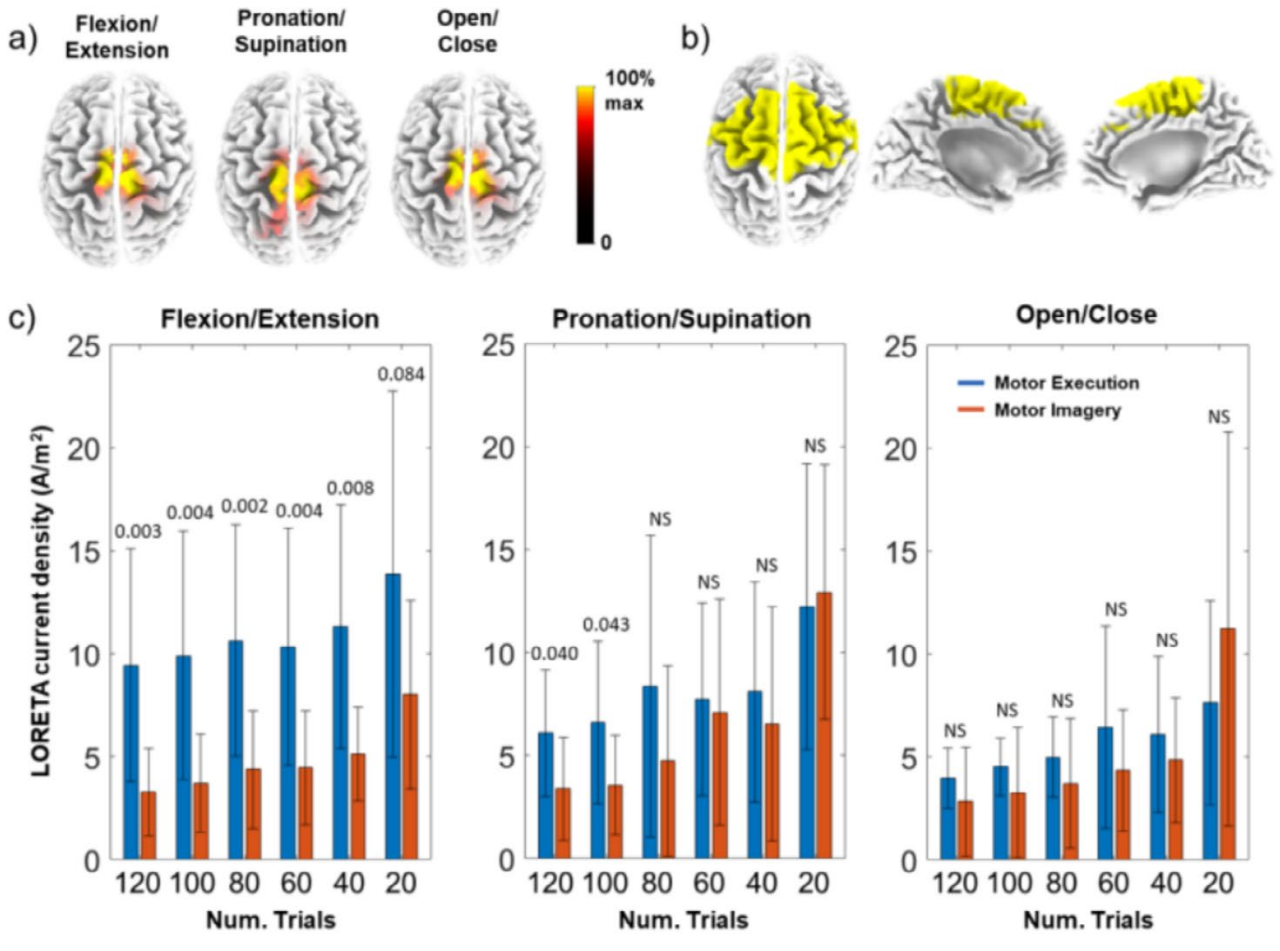
(a) LORETA maps for MRCP peak of MI dataset and for each movement category; (b) LORETA maps of BA6 and (c) Barplots corresponding to LORETA values when reducing the number of trials for ME (blue) and MI (red) datasets. *p*‐values ≤ 0.1 of ME and MI differences are depicted (not significant (NS) corresponds to *p*‐value > 0.1).

MRCP peak tomographic sources were measured using sLORETA for ME and MI dataset and each movement class. Regardless of the movement class, both ME and MI exhibited the greatest current density value in the MRCP peak in Brodmann area 6 (BA6, Medial Frontal Gyrus, Frontal Lobe). Figure 6a shows, as an example, the average MRCP peak brain source localization across subjects for the three movement classes, considering the whole number of free‐artifact trials. A ROI defined by all the voxels comprising the BA6 (see Figure 6b) was used to determine statistical differences in the brain activation patterns related to actual and imagery movements. No significant voxels were observed outside the BA6 between ME and MI after the correction for multiple comparisons. Paired statistical *t*‐tests for averaged current density values in BA6 between ME and MI conditions were performed for each movement class, considering a different number of trials for MRCP averaging. The BA6 sLORETA values, displayed in Figure 6c, demonstrated a slightly increasing trend as the number of trials decreased, for both ME and MI, and for all movement classes. Statistical significance between BA6 scores linked to ME and MI was no longer observed for flexion/extension and especially for pronation/supination, when the number of trials was reduced (up to 60 and 100 trials, respectively).

## 4 Discussion

The study of motor‐related cortical activity during ME and MI has been widely explored (Debarnot et al. 2021; Ibáñez et al. 2014; Monge‐Pereira et al. 2017; Mulder 2007), with MI being particularly useful in brain‐computer interface (BCI) technologies and as a therapeutic tool for brain plasticity and functional restoration in motor disorder patients (Adams et al. 2018; Hochberg et al. 2006; Kübler and Birbaumer 2008; Mulder 2007; Zich et al. 2015). MRCP and ERD are key motor‐related cortical activities used to assess neuromotor pathologies and track motor rehabilitation progress (El Nahas et al. 2022; Fong et al. 2021; Gong et al. 2013; Heida et al. 2014; Houdayer et al. 2014; Stępień et al. 2011). However, since MRCP and ERD require averaging multiple trials, fatigue can affect data accuracy, leading to unreliable results (Boksem et al. 2005; Jacquet, Lepers, et al. 2021; Jacquet, Poulin‐Charronnat, et al. 2021). This study aimed to investigate how the number of trials impacts MRCP and ERD analysis during ME and MI tasks, highlighting the importance of trial quantity in ensuring accurate motor‐related cortical activity identification and proper statistical analysis of the differences between conditions.

Multiple works in the literature have analyzed the similarities and divergences between the neural mechanisms underlying both ME and MI processes. In our study, differences in motor‐related cortical activity between ME and MI have been revealed in concordance with previous studies. However, a quantitative study in terms of statistical significance and using different approaches (MRCP, ERDs, topographic and tomographic maps) has been performed for the first time considering a different number of trials. For instance, MRCP amplitude was greater for ME than MI during all movement classes studied (Nascimento et al. 2006; Ofner et al. 2017). Similar results were obtained for ERD mu and beta, where ME reached higher desynchronization than MI, as it has been seen before (Van der Lubbe et al. 2021). Differences in their values for ME and MI between movement categories appeared in line with previous research (Jankelowitz and Colebatch 2002; Pfurtscheller and Lopes da Silva 1999). Moreover, our analysis of the interaction between motor features and movement classes indicated a statistically significant difference between ME and MI that was more pronounced for the movements with the highest motor‐related cortical activity amplitude values.

Consistent with previous research, reducing the number of trials used to analyze motor features, such as MRCP peak and the ERD min in mu and beta bands, led to a higher degree of intersubject variability (Borràs et al. 2022; Goldsworthy et al. 2016). While both ME and MI conditions exhibited a similar trend when reducing the number of trials, MI demonstrated greater variability and differences in motor features across all movement classes. This suggests that decreasing the number of trials has a more significant impact on the determination of MI motor features being more sensitive than ME.

The analysis of the statistical differences between ME and MI underscored the significance of determining the appropriate number of trials based on the specific motor feature or movement being studied. Specifically, in certain motor features or movement classes, the differences between ME and MI lost their significance as the number of trials decreased. For instance, the ERD min in the beta band showed statistical differences regardless of the number of trials or movement categories, whereas the ERD min in mu only reached statistical significance for all movements when at least 60 trials were included. Additionally, the MRCP peak exhibited a similar pattern to that of the minimum ERD in the beta band, with the exception of the hand open/close movement, which only showed statistical differences between actual and imagery after averaging 70 trials or more. These findings were supported by topographic maps, where ME and MI presented comparable scalp distributions for all movements and number of trials considered in the averaging. Nonetheless, as the number of trials decreased, statistical significance between ME and MI declined, particularly for the minimum ERD in the mu band. The key differences between ME and MI for all analyzed movements were primarily located in the regions with higher feature values: specifically, the motor cortex (around Cz) in the case of the MRCP peak (Claflin et al. 2015; Colebatch 2007; Rektor et al. 2003), and the contralateral sensorimotor area (around ERD beta and mu ROIs) for the ERD min (Pfurtscheller and Lopes da Silva 1999; Tariq et al. 2020).

In a previous work, the effect of the number of trials on the lateralized readiness potential (LRP) was assessed (Boudewyn et al. 2018). The LRP is an ERP related to motor preparation and control and is calculated by the difference of ipsilateral and contralateral central site electrodes when an individual is prepared to make a movement with one of their hands. As the number of trials increased, the statistical power remained at a minimum or maximum level for small and large group differences, respectively. Conversely, for moderate group differences, the power escalated significantly with an increase in the number of trials. Our findings support this conclusion. Specifically, variability appears to be the driving factor. When we decrease the number of trials for motor‐related cortical activity averaging, intersubject variability increases. For small or large group differences, variability has little effect on statistical significance (e.g., minimum ERD in the beta band shown in Figure 4). However, for intermediate group differences, a reduction in trial number results in a decrease in statistical power due to increased variability (see Figure 4).

Our results also revealed that the impact of the number of trials on the observed tomographic source differences between actual and imagined movements was not uniform across all movement classes. Rather, elbow flexion/extension was more sensitive to changes than the other movements analyzed when reducing the number of trials. The main differences were located on BA6, which corresponded with the region of highest current density (Hanakawa et al. 2002; Picard and Strick 1996). Statistical differences between ME and MI were found for flexion/extension across all the number of trials. However, whereas the LORETA current density values did not show a significant *p*‐value between executing and imaging movement of opening/closing the hand, differences were statistically significant between ME and MI pronation/supination when averaging 80 trials or more.

While our study investigated the effect of trial number on averaged motor‐related cortical activity using a Monte Carlo reduction approach, it is important to consider other analysis techniques, particularly when dealing with less pronounced MRCPs, such as those in stopped movements or motor attempts (Galdo‐Alvarez et al. 2016). In such cases, a higher number of trials for averaging may be necessary due to a lower signal‐to‐noise ratio. Furthermore, methods like single‐trial analysis with epoch alignment based on response delay (Syrov et al. 2024) can provide insights into the temporal dynamics of MRCPs in relation to response speed, an aspect not directly addressed by our averaging‐focused methodology. Similarly, response‐time binning (Poli et al. 2010) offers a way to improve averaging quality by accounting for reaction time variability. Our study's focus on the impact of trial quantity provides valuable information for experiment design, particularly regarding fatigue and task duration. However, future research could benefit from exploring how the selection and alignment of trials based on response characteristics influence the analysis of motor‐related cortical activity, potentially offering complementary insights into the neural mechanisms of motor control and imagery.

One limitation of our approach is that trial selection was performed randomly rather than considering the temporal evolution of the features throughout the session. While this strategy aimed to avoid biases related to trial order, it does not account for potential fatigue‐related effects over time. Future analyses could explicitly examine the temporal progression of MRCP and ERD features to assess the influence of fatigue on brain activity patterns.

## 5 Conclusion

This study highlights the importance of determining the appropriate number of trials according to the specific motor feature and movement being analyzed for comparative purposes between two groups. Our results revealed that certain differences between motor and imagery movement, which had been previously reported in the literature (Miller et al. 2010; Nascimento et al. 2006), lost their significance when the number of trials was reduced. Significant statistical differences between ME and MI of elbow and forearm related movements have been observed in terms of feature values. Specifically, MRCP peak and ERD beta min showed significant differences after 20 trials, while ERD mu min required at least 60 trials to achieve statistical significance. On the other hand, these differences between ME and MI for hand open/close movement are statistically significant after 20 trials for ERD beta min, 60 for ERD mu min, and 70 for MRCP peak.

These findings have significant implications for motor rehabilitation studies (Adams et al. 2018; Charbonnier et al. 2024), where precise motor feature identification is critical. Therefore, determining the optimal number of trials for analyzing motor features, whether for actual or imagined movement, is essential. This consideration should be tailored to the specific movement type and motor feature under research. Researchers must strike a balance between the number of trials needed to ensure statistical significance and the risk of mental and physical fatigue that may arise in participants, especially in lengthy experimental sessions (Guillot et al. 2009; Jeannerod 2001). As recording sessions can be time‐consuming, researchers must ensure reliable results can be achieved within a reasonable timeframe.

Finally, since these results were derived from healthy subjects performing both ME and MI on consecutive days, future research should explore the impact of trial numbers, comparing actual and imagery movements, in clinical populations with motor disorders, as well as investigate other movement types or different experimental paradigms.

## Author Contributions

**Marta Borràs:** methodology, formal analysis, writing – review and editing, writing – original draft, conceptualization, investigation. **Sergio Romero:** conceptualization, funding acquisition, supervision, writing – original draft, writing – review and editing, formal analysis, investigation. **Leidy Y. Serna:** conceptualization, formal analysis, writing – review and editing, writing – original draft. **Joan F. Alonso:** writing – original draft, writing – review and editing, formal analysis. **Alejandro Bachiller:** methodology, formal analysis, writing – review and editing, writing – original draft. **Miguel A. Mañanas:** conceptualization, writing – original draft, writing – review and editing, funding acquisition, supervision. **Mónica Rojas:** conceptualization, writing – original draft, writing – review and editing, methodology, formal analysis.

## Conflicts of Interest

The authors declare no conflicts of interest.

## Data Availability

The data that support the findings of this study are openly available in BNCIHORIZON2020 at https://bnci‐horizon‐2020.eu/database/data‐sets, reference number 001‐2017.
